# Development and validation of a nutrition risk screening for patients with childhood cancer in Brazil (NUTRICCAN)

**DOI:** 10.1002/ncp.70076

**Published:** 2025-12-04

**Authors:** Cristiane Ferreira Marçon, Carolina Araújo dos Santos, Fernanda Luisa Ceragioli Oliveira

**Affiliations:** ^1^ Postgraduate Program in Nutrition, Federal University of São Paulo São Paulo Brazil; ^2^ Department of Nutrition and Health Federal University of Viçosa Viçosa Minas Gerais Brazil; ^3^ Department of Pediatrics Discipline of Pediatric Nutrology, Escola Paulista de Medicina Federal, University of São Paulo São Paulo Brazil

**Keywords:** life cycle, nutrition, nutrition assessment, oncology, pediatrics, research and diseases

## Abstract

**Background:**

Diagnosing malnutrition in patients with pediatric cancer is challenging because tumor masses can interfere with anthropometric measurements. STRONGkids considers cancer a general risk factor, whereas Screening Tool for Childhood Cancer (SCAN) classifies patients as at risk or not, potentially missing those who need nutrition monitoring. This study describes development and validation of a new nutrition risk screening tool for childhood cancer in Brazil.

**Methods:**

Nutrition Risk Screening for Childhood Cancer (NUTRICCAN) underwent online expert content validation. Twelve nutritionists applied it to oncology inpatients (0–19 years old) at the Pediatric Oncology Institute at Federal University of São Paulo from June to August 2024. Nutrition risk was assessed using NUTRICCAN, STRONGkids, and SCAN. Logistic regression identified screening variables associated with malnutrition (body mass index‐for‐age *z* score < −2, mid‐upper arm circumference [MUAC] <5^th^ percentile, calf circumference below cutoff, or met any of the criteria for malnutrition). Receiver operating characteristic analysis determined cutoff scores, and sensitivity/specificity analyses allowed comparisons among instruments.

**Results:**

Patients not receiving intensive care were almost three times more likely to have an inadequate MUAC (odds ratio [OR], 4.505; 95% confidence interval [CI], 1.446–14.033; *P* = 0.009). Low socioeconomic status or caregiver education increased the risk of malnutrition (OR, 2.845; 95% CI, 1.070–7.566; *P* = 0.036). Dietitians’ subjective assessments were associated with a fourfold increased risk of malnutrition. NUTRICCAN was 70% accurate (area under the curve, 0.701; 95% CI, 0.617–0.785), outperforming the other tools.

**Conclusion:**

NUTRICCAN better stratifies nutritional risk, considering clinical and socioeconomic factors, and may allow for more targeted interventions, especially in resource‐limited settings.

## INTRODUCTION

Patients with cancer are at high risk for malnutrition because of a combination of factors, including increased energy requirements from tumor catabolism and decreased food intake from treatment side effects such as nausea, vomiting, taste changes, and loss of appetite.^1^ Infants, children, and adolescents are at an even greater risk because of their growth needs and the essential nutrients necessary for brain development in children <5 years old. They are also at risk because of physiological and inflammatory hormonal changes.^2^, ^3^

Diagnosing malnutrition in this population is challenging because tumor masses can interfere with measuring and interpreting anthropometric parameters. Because there is no gold standard method for determining nutrition status in children with cancer, objective and subjective assessments by experienced professionals are required.^2^

Because malnutrition negatively affects patients and their treatment, it is essential to identify those at higher nutrition risk for early intervention.^4^ To date, only one screening tool has been translated and validated for the Brazilian pediatric population: STRONGkids.^5^, ^6^ This method considers a cancer diagnosis a general risk factor without distinguishing between cancer types or treatment methods, even though the literature suggests different nutrition outcomes for each.^1^ In recent years, new screening tools have been developed for the pediatric oncology population. Examples include the Screening Tool for Childhood Cancer (SCAN),^7^ which has been translated into Portuguese,^8^ and the Nutrition Risk Screening for Pediatric Cancer.^9^ Although SCAN is oncology specific, it only classifies patients as either at risk or not. This implies that some patients may not require nutrition monitoring.

This study aims to describe the development process and concurrent validation of a nutrition risk screening tool for infants, children, and adolescents with cancer within the Brazilian sociocultural context.

## METHODS

### Screening tool development and content validation

A literature review of available pediatric nutrition screening tools was conducted.^5^, ^7^, ^9^, ^10^, ^11^, ^12^ Based on this analysis, the authors formulated and divided relevant questions into domains or topic areas along with scoring criteria.^13^ An online form containing these questions was submitted to an expert panel of physicians and nutritionists from specialized centers for individual content validation on a dichotomous scale (“agree” and “disagree”) and a Likert scale (1 is not clear or representative, 2 is somewhat clear or representative, 3 is fairly clear or representative, and 4 is very clear or representative).

Agreement rate (number of raters agreeing/total number of raters × 100) and content validity index (CVI) (number of responses rated 3 or 4/total number of responses) were calculated. Items with an agreement rate <90% or a CVI <0.8 were discussed in two online meetings with the committee to resolve discrepancies and finalize the tool, named Nutrition Risk Screening for Childhood Cancer (NUTRICCAN).

### Pilot test

A pilot test was conducted at the Pediatric Oncology Institute at the Federal University of São Paulo (IOP/GRAACC) in São Paulo, Brazil to determine concurrent validation and agreement with other screening tools. Twelve nutritionists were trained to use the tool with patients in oncology aged 0–19 years old in an inpatient setting. Patients admitted during the first week of June 2024 were included in the study, with the exception of those in intensive care units or undergoing hematopoietic stem cell transplantation.

### Data collection

After receiving approval from the Ethics and Research Committee of the Federal University of São Paulo (CAAE: 65610622.2.0000.5505), data collection took place from June to August of 2022. Informed consent was obtained for the participating patients. The variables included sex, age (in years), and cancer diagnosis. Nutrition risk was assessed using NUTRICCAN, as well as STRONGkids and SCAN. The Brazilian Portuguese translated and culturally adapted versions of the STRONGkids and SCAN tools were used.^6^, ^8^

Weight, height, mid‐upper arm circumference (MUAC), and calf circumference (CC) were measured according to the recommendations of the Brazilian Society of Pediatrics.^14^ Body mass index (BMI) for age was calculated using Anthro version 3.2.2 for children <5 years old and Anthro Plus version 1.0.4 for children >5 years old.^15^, ^16^ Indices were expressed as *z* scores, and nutrition status was classified as follows: malnutrition (*z* score ≤ −2), eutrophic (*z* score > −2 and < 1), or overweight (*z* score ≥ 1). A BMI‐for‐age cutoff of *z* score ≤−1 was used to define patients at nutrition risk, as suggested by American Society for Parenteral and Enteral Nutrition (ASPEN) guidelines and the Brazilian Pediatric Oncology Nutrition Survey (2021).^17^, ^18^ MUAC was categorized into percentiles, following the World Health Organization (WHO) guidelines (2006)^15^ for children <5 years of age and Frisancho^19^ for older children. A percentile <5 was considered a marker of malnutrition. CC was classified according to age‐specific and sex‐specific cutoff points,^20^ with values below the cutoff indicating malnutrition.

### Statistical analysis

Microsoft Excel, SPSS (version 27), and Jamovi (version 2.3.28) software programs were used for data tabulation and statistical analysis. Sex, cancer diagnosis, risk, and nutrition status were presented as absolute (*n*) and relative (%) frequencies. Age, BMI‐for‐age, MUAC, and CC were presented as the mean and standard deviation or the median and interquartile range (IQR), depending on the results of the Shapiro‐Wilk test for normality. Group differences were evaluated using chi‐square test, Student *t* test, or Mann‐Whitney tests. Statistical significance was set at *p* < 0.05.

Logistic regression models were used to identify screening variables associated with malnutrition for the following outcomes: BMI‐for‐age *z* score <−2, MUAC below the fifth percentile, CC below the cutoff, or at least one altered parameter. In the univariate analysis, each variable was tested individually by estimating the odds ratio (OR) and its 95% confidence interval (CI), with a *p* value of <0.20 indicating statistical significance. In the multivariate analysis, all variables were included if the collinearity principles were met (variance inflation factor <5 and tolerance >0.8).

A receiver operating characteristic (ROC) curve analysis was performed to determine cutoff scores and categorize risk strata. First, the ROC analysis identified the high‐risk stratum for malnutrition with greater precision. The area under the curve (AUC) was used as a measure of discrimination, and the cutoff point that balanced sensitivity and specificity best was selected. Malnutrition was defined as the presence of at least one altered anthropometric parameter (BMI‐for‐age *z* score ≤–2, MUAC below the fifth percentile, or CC below the age‐specific and sex‐specific cutoff), which served as the reference standard for the ROC analysis. A second ROC analysis was performed to determine the cutoff for identifying those without malnutrition and to establish the low‐risk and intermediate‐risk strata. Sensitivity and specificity analyses were also performed for SCAN and STRONGkids, enabling comparisons between the instruments based on identical outcome measures.^21^, ^22^

Spearman rho test was used to analyze the correlation between the proposed screening tool scores and the SCAN and STRONGkids scores. Correlations were considered low when *ρ* ≤ 0.50, moderate when *ρ* < 0.70, and high when *ρ* ≥ 0.70.^23^, ^24^ Kendall's tau test was used to assess the agreement between the nutrition risk classifications of the three tools.^25^

## RESULTS

### Screening tool development and content validation

The proposed tool includes 15 variables divided into seven domains: (1) cancer diagnosis, (2) current treatment, (3) sociodemographic and clinical factors, (4) gastrointestinal tract changes, (5) dietary intake, (6) weight loss, and (7) subjective professional nutrition assessment.

Treatment centers and professionals were invited to participate in the validation of the screening tool. Those who completed the online evaluation by the deadline formed the expert panel, consisting of nine nutritionists and four pediatric oncologists (one from the North region, one from the Northeast, one from the Midwest, eight from the Southeast, and two from the South).

Table 1 shows the agreement rates and CVI of each evaluated item, as well as the suggested text changes from the committee.

**Table 1:** Agreement rates and CVI for screening items and text modification proposals.

	Clarity		Representativeness		
Initial proposal	Agreement, %	CVI	Agreement, %	CVI	Final text version
Domain 1—Diagnosis: Which group best describes the patient's oncological diagnosis?	84.62*	–	92.31	–	No changes
Item 1.1: Tumors with extensive abdominal masses Wilms tumor: stage III and IV Neuroblastoma: stage III and IV Abdominal or pelvic rhabdomyosarcomas Burkitt‐type NHL in the abdominal region	92.31	1.0000	92.31	0.8462	Tumors with extensive abdominal masses examples: Wilms tumor–stage III and IV, neuroblastoma–stage III and IV, abdominal or pelvic rhabdomyosarcomas, Burkitt‐type NHL in the abdominal region, among others.
Tumors in the head and neck region: Head and neck carcinomas (oral cavity, larynx, pharynx, and esophagus) Rhabdomyosarcomas (oral cavity, larynx, pharynx, and esophagus) NHL in the head and neck region Medulloblastoma diencephalic tumors, Ewing sarcoma, and osteosarcoma relapses of leukemias and lymphomas					Tumors in the head and neck region (at any stage) examples: Head and neck carcinomas (oral cavity, larynx, pharynx, and esophagus), rhabdomyosarcomas (oral cavity, larynx, pharynx, and esophagus), NHL in the head and neck, among others. Medulloblastoma diencephalic tumors, Ewing sarcoma, osteosarcoma relapses of leukemias and lymphomas or with comorbidities (eg, pancreatitis)
Item 1.2: Other solid tumors and acute leukemias or lymphomas without complications	84.62*	0.9231	76.92*	0.9231	Other solid tumors examples: Wilms tumor–stage I and II, neuroblastoma–stage I and II, among others. Acute leukemias or lymphomas without complications
Item 1.3: Diseases in remission, during maintenance treatment, and craniopharyngioma	92.31	0.9231	92.31	0.9231	No changes
Domain 2—Treatment: Is the current treatment intensive and may lead to nutrition impairment? If any of the items are true, score:	92.31	–	100	–	No changes
Item 2: First cycle of chemotherapy; recent use (in the last 15 days) or planned use for the coming days, of chemotherapeutic agents with emetic potential or intestinal toxicity:	92.31	0.9231	100	1.0000	No changes
eg, cisplatin, cyclophosphamide, methotrexate, cytarabine, 5‐fluorouracil, or irinotecan					
Treatments involving (in the last 3 months or planned for the next 7 days): Head and neck surgeries (except neurosurgery) or abdominal surgeries Pelvic, abdominal, head and neck, or cranial radiotherapy (with or without neuroaxis)					
Postoperative complications (consider up to 15 days after surgery)					
Posthematopoietic stem cell transplant complications (eg, GVHD)					
Domain 3—Sociodemographic and clinical factors: Does the patient present any of the risk factors below? Score each true item:	92.31	–	100	–	No changes
Item 3: Age group: Infants <1 year old	92.31	1.0000	100	0.9231	No changes
Item 4: Age group: Early childhood (1 to 3 years old) or adolescence (rapid growth phase)	92.31	0.9231	100	1.0000	No changes
Item 5: Low socioeconomic status (eg, reliance on public healthcare system) or low educational level of caregivers	38.46*	0.4615*	53.85*	0.6154*	Low socioeconomic status (<1 minimum wage) or low educational level of caregivers (<8 years of schooling)
Item 6: Pain that interferes with food intake (regardless of location)	92.31	0.9231	84.62*	0.8462	Pain (regardless of location) that interferes with food intake
Item 7: Respiratory discomfort that interferes with food intake	100	1.0000	92.31	1.0000	No changes
Item 8: Hospitalization duration >15 days	100	1.0000	100	0.9231	No changes
Item 9: Readmission within 7 days	100	1.0000	100	1.0000	No changes
Domain 4—Gastrointestinal alterations: Does the patient have gastrointestinal alterations in the last 24 h? Score each true item:	92.31	–	92.31	–	No changes
Item 10: Diarrhea: ≥5 liquid stools in the last 24 h or fecal incontinence or dehydration	92.31	0.9231	76.92*	0.7692*	Diarrhea: ≥5 liquid stools in the last 24 h or any frequency with clinical repercussions (eg, dehydration)
Item 11: Mucositis: Worsening of lesions in the last 24 h, regardless of grade	76.92*	0.8462	69.23*	0.8462	Mucositis: Presence of oral mucositis that, regardless of grade, hinders food intake
Item 12: Vomiting: ≥3 episodes of vomiting in the last 24 h	92.31	0.8462	84.62*	0.9231	Vomiting: ≥3 episodes of vomiting in the last 24 h or any frequency with clinical repercussions (eg, dehydration)
Domain 5—Food intake: Has the patient experienced changes in their food intake in the last few days? Score each true item:	92.31	–	92.31	–	No changes
Item 13.1: Fasting ≥72 h without nutrition support	84.62*	0.9231	92.31	0.9231	Oral or enteral fasting ≥72 h without nutrition support (enteral or parenteral)
Item 13.2: Anorexia: Complete lack of appetite in the last 48 h	92.31	1.0000	92.31	1.0000	No changes
Item 13.3: <2 complete meals (about 50% of daily needs) in the last 3 days or regular food intake (about 70% of daily needs) for >1 week or use of enteral feeding tube with intake below the prescribed nutrition requirements	76.92*	0.8462	84.62*	0.9231	<2 complete meals (about 50% of daily needs) in the last 3 days or regular food intake (about 70% of daily needs) for ≥1 week or more or use of enteral feeding tube with intake below the prescribed volume
Item 13.4: Not applicable (fasting for examination or surgery or full enteral diet administration)	92.31	0.9231	92.31	0.9231	No changes
Domain 6—Weight loss: Do the parents or caregivers (or the patient themselves) notice that the patient has lost weight in the last month?	76.92*	–	100	–	No changes
Item 14.1: Yes, there was significant and very noticeable weight loss	84.62*	0.7692*	69.23*	0.6923*	No changes
Item 14.2: Yes, there was slight weight loss	76.92*	0.6923*	69.23*	0.6923*	Yes, there was slight or mild weight loss (eg, clothes became looser)
Item 14.3: Not sure if weight was lost	100	1.0000	100	1.0000	No changes
Item 14.4: Did not lose weight	100	1.0000	100	1.0000	No changes
Domain 7—Subjective nutrition assessment by the professional: Does the patient show signs of nutrition compromise based on the overall patient assessment?	92.31	–	100	–	No changes
Item 15.1: The patient shows clear signs of muscle and/or fat depletion, cachexia, sarcopenia, or a record of weight loss >2% documented in the medical record, leaving no doubt about the presence of malnutrition	100	1.0000	100	1.0000	No changes
Item 15.2: I have doubts whether the patient shows nutrition compromise (because of hyperhydration, for example)	100	1.0000	100	1.0000	No changes

Abbreviations: CVI, Content Validity Index; GVHD, graft‐vs‐host disease; NHL, non‐Hodgkin lymphoma. *Screening itens with agreement rate <90% or CVI <0.8.

### Screening tool pilot testing: Concurrent validation

A total of 454 screenings were performed. Six were excluded because of incomplete data, and 27 were excluded because the patients had completed cancer treatment. After excluding duplicate screenings, the final sample consisted of 152 patients. The majority were boys (56.6%), with a median age of 6.73 years old (IQR, 3.15–11.8) and a higher prevalence of central nervous system tumors (27%) and hematologic malignancies (25.7%) (Table 2).

**Table 2:** Demographic data and anthropometric measures of the pediatric cancer population.

	Girls	Boys	*P* value
Sex, *n* (%)	66 (43.4)	86 (56.6)	0.123^a^
Age, median (IQR), y	6.86 (3.30–11.2)	6.62 (3.64–12.4)	0.742^b^
Type of cancer, *n* (%)			0.979^a^
Central nervous system	17 (11.2)	24 (15.8)	
Hematological	17 (11.2)	22 (14.5)	
Abdominal tumors^c^	13 (8.6)	19 (12.5)	
Bone tumors	8 (5.3)	9 (5.9)	
Others^d^	11 (7.2)	12 (7.9)	
BMI, mean (SD), kg/cm^2^	17.6 (±4.65)	17.4 (±3.79)	0.800^e^
BMI‐for‐age, mean (SD), *z* score	0.09 (±1.50)	0.04 (±1.52)	0.926^e^
MUAC, median (IQR), cm	17 (15–22)	17 (15–22.7)	0.805^b^
CC, median (IQR), cm	22 (19–28.5)	22.5 (19–28.5)	0.672^b^

Abbreviations: BMI, body mass index; CC, calf circumference; IQR, interquartile range; MUAC, mid‐upper arm circumference; SD, standard deviation.

^a^ Pearson chi‐square test.

^b^ Mann‐Whitney *U* test.

^c^ Renal, hepatic tumors, neuroblastomas, germ cell tumors, or immature teratomas of the reproductive system (testicle or ovaries).

^d^ Ocular tumors, sarcomas (except bone Ewing sarcoma), or carcinomas.

^e^ Student *t* test.

Nutrition status varied according to the assessment method. According to the BMI‐for‐age *z* score, the sample had a mean of 0.05 (±1.51 SD). A total of 7% (*n* = 11) were classified as undernourished, 68% (*n* = 103) as eutrophic, and 25% (*n* = 38) as overweight. Nutrition risk, defined as a BMI‐for‐age *z* score of ≤−1, occurred in 25.7% (*n* = 39) of the sample. Using MUAC, 24.8% (*n* = 37) were malnourished, whereas 40.1% (*n* = 61) were below the CC cutoffs by age and sex. Combining all parameters, 45.4% of the sample (*n* = 69) met at least one criterion for malnutrition.

The final NUTRICCAN score ranged from 1 to 26 points, with a median of 11 (IQR, 6–16). Patients with a score of ≥10 on the NUTRICCAN were identified as being at nutrition risk, which corresponds to the high‐risk category. The cutoff point for high risk was determined through ROC curve analysis by considering the probability of a malnutrition diagnosis based on the studied anthropometric parameters as the outcome. The full screening tool and scoring system are available in the Supporting Information.

Of the cases, 19.7% (*n* = 30) were classified as low risk (1–4 points), 25.6% (*n* = 39) as intermediate risk (5–9 points), and 54.6% (*n* = 83) as high risk (≥10 points). SCAN classified 51.3% (*n* = 78) of cases as nutritionally at risk, whereas STRONGkids classified none as low risk, 67.1% (*n* = 102) as intermediate risk, and 32.8% (*n* = 50) as high risk.

Logistic regression analyses were conducted to identify which variables in the screening tool were associated with each anthropometric marker of malnutrition. In multivariate models, patients not receiving intensive treatment were significantly more likely to have low MUAC (OR, 4.505; 95% CI, 1.446–14.033; *P* = 0.009). Low socioeconomic status and low caregiver education were associated with an increased risk of malnutrition when assessed by MUAC (OR, 2.45; 95% CI, 1.2–5.2; *P* = 0.02) and CC (OR, 2.87; 95% CI, 1.3–6.2; *P* = 0.01). Reduced food intake (item 13.3) was significantly associated with malnutrition when assessed by CC (OR, 2.07; 95% CI, 1.1–4.0; *P* = 0.03), although this significance was not sustained in the combined model. The nutritionist's subjective assessment was the most consistent predictor of all outcomes, with ORs ranging from 4.52 to 41.0 in multivariate models. Detailed regression results for each anthropometric parameter are provided in Tables S1–S4.

According to feedback from the dietitians who applied the tool during the pilot test, the NUTRICCAN was considered easy and quick to administer, without interfering with their routine. The professionals also reported no doubts regarding the interpretation of the questions by themselves, the patients, or their caregivers.

### Screening tool pilot testing: Agreement with other screening tools

**Figure 1:**
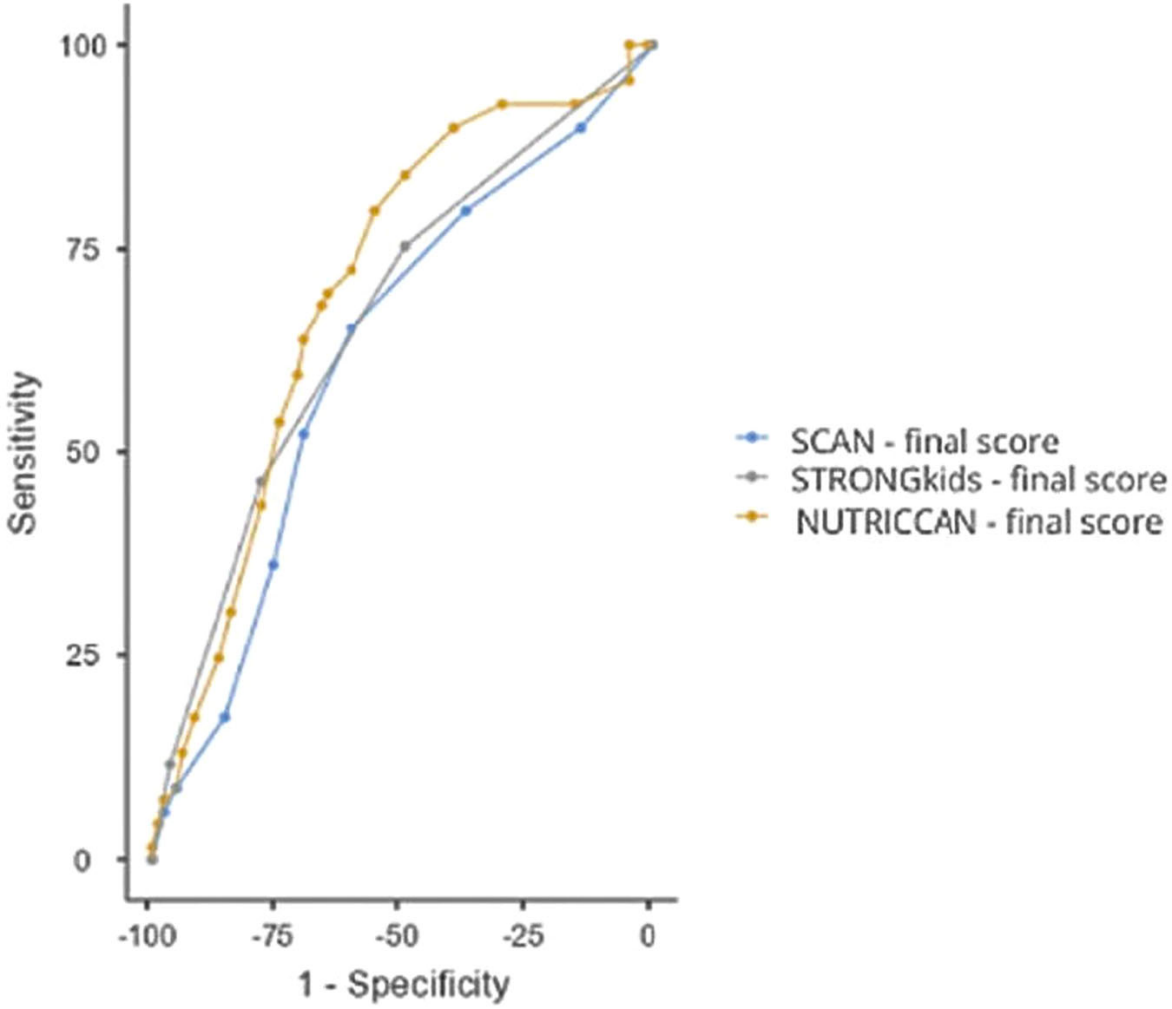
Receiver operating characteristic curves of the screening tools for identifying malnutrition. NUTRICCAN, nutrition risk screening for childhood cancer; SCAN, Screening Tool for Childhood Cancer.

ROC curve analyses showed that the newly proposed tool had an accuracy of 70% (AUC, 0.701; 95% CI, 0.617–0.785) in identifying malnutrition based on any anthropometric criterion. This result outperformed those of STRONGkids (AUC, 0.664; 95% CI, 0.577–0.751) and SCAN (AUC, 0.621; 95% CI, 0.531–0.711) (Figure 1). The ROC analysis was based on the presence of at least one anthropometric indicator of malnutrition, which was used as the reference standard. The optimal cutoff score for high risk in NUTRICCAN was 10 or greater, with a sensitivity of 72.46% (95% CI, 0.604–0.825) and a specificity of 60.24% (95% CI, 0.489–0.708) for detecting malnourished patients (Table 3).

**Table 3:** Accuracy and performance of the screening tools in identifying malnutrition, based on any anthropometric diagnostic criteria (body mass index‐for‐age *z* score below −2 and/or mid‐upper arm circumference below the fifth percentile and/or calf circumference below the cutoff).

Tool	Cutoff	Sensitivity, % (95% CI)	Specificity, % (95% CI)	PPV, % (95% CI)	NPV, % (95% CI)	AUC, % (95% CI)
NUTRICCAN	≥10	72.46 (0.604–0.825)	60.24 (0.489–0.708)	60.24 (0.489–0.708)	72.46 (0.604–0.825)	0.701 (0.617–0.785)
SCAN	≥3	65.22 (0.528–0.763)	60.24 (0.489–0.708)	57.69 (0.460–0.688)	67.57 (0.557–0.780)	0.621 (0.531–0.711)
STRONGkids	≥4	46.38 (0.343–0.588)	78.31 (0.679–0.866)	64 (0.492–0.771)	63.73 (0.536–0.730)	0.664 (0.577–0.751)

Abbreviations: AUC, area under the curve; CI, confidence interval; NPV, negative predictive value; NUTRICCAN, nutrition risk screening for childhood cancer; PPV, positive predictive value; SCAN, Screening Tool for Childhood Cancer.

The tool performed less satisfactorily when predicting the absence of malnutrition based on anthropometric parameters. With a cutoff of ≤5 for low risk, the sensitivity was 86.5% (95% CI, 0.639–0.980), and the specificity was 11% (95% CI, 0.086–0.252), with an AUC of 0.365 (95% CI, 0.179–0.551).

Spearman correlation coefficient (rho) showed a strong correlation between NUTRICCAN scores and SCAN (*ρ* = 0.839; 95% CI, 0.782–0.881; *P* < 0.001) and STRONGkids (*ρ* = 0.811; 95% CI, 0.746–0.860; *P* < 0.001). Concordance analysis revealed higher agreement between NUTRICCAN and SCAN (*τ* = 0.726; 95% CI, 0.672–0.773; *P* < 0.001) than between NUTRICCAN and STRONGkids (*τ* = 0.561; 95% CI, 0.484–0.630; *P* < 0.001).

## DISCUSSION

Pediatric screening tools typically treat cancer as a single pathological entity, ignoring differences in diagnoses and treatment modalities. For example, when used in a specific oncology treatment center, the STRONGkids tool loses its ability to stratify nutrition risk into levels. Although SCAN is specific to the oncology population, it establishes a single cutoff point at which patients are considered at nutrition risk. This implicitly suggests that some patients are not at nutrition risk, which is a controversial conclusion given the nutrition vulnerability imposed by cancer physiology and treatment.^26^

Malnutrition may be present at the time of diagnosis and tends to worsen during treatment. Obesity is more common among survivors but can also be observed early on in patients with brain tumors or who are receiving high‐dose steroids.^27^ Similar diagnoses may require different treatment approaches, resulting in different nutrition outcomes. Patients undergoing treatment for high‐risk acute lymphoblastic leukemia are more likely to develop protein‐energy malnutrition than patients undergoing treatment for standard or low‐risk conditions.^28^ In this study, intensive treatment was inversely associated with reduced MUAC. Poor nutrition status may limit patients’ tolerance of more aggressive therapies because of a higher prevalence of toxicities. Malnutrition can impair tolerance to antineoplastic treatment, negatively impact overall and event‐free survival, increase the risk of infection, and affect quality of life.^27^

Because of social, cultural, and economic differences, instruments developed and validated in high‐income countries may behave differently in low‐income regions.^29^ The presence of social vulnerability factors, such as low family income and caregiver education, was a significant predictor of malnutrition in this sample, but this aspect is not examined in SCAN or STRONGkids. In Colombia, Pinzón‐Espitia et al found that over half of the children at an oncology hospital experienced food insecurity, and 76% were at nutrition risk, as measured by SCAN.^30^ Economic resource scarcity can significantly increase the risk of inadequate dietary intake, both quantitatively and qualitatively. Belle et al found a twofold increased risk of overweight and obesity in adult survivors of acute lymphoblastic leukemia with low family income.^31^ Including socioeconomic factors in nutrition risk screening in Brazil is important because it broadens the professional view of the food environment to which these patients are exposed.

Although nutrition classification based on anthropometric parameters using weight and height alone is common in pediatrics, it is insufficient for accurately measuring malnutrition. During nutrition transitions, malnutrition and being overweight may coexist, which emphasizes the importance of assessing nutrition status by focusing on body composition.^1^ Subjective nutrition assessment tools, such as ANPEDCancer, correlate well with body composition assessments and can identify nutrition risk. However, these tools require more time to administer because they are designed to make a nutrition diagnosis and include anthropometric, physical, and dietary evaluations.^32^ MUAC is recognized as a simple, inexpensive method with a satisfactory correlation with lean mass stores,^1^, ^27^ as is CC.^20^ In this study, MUAC and CC were used alongside BMI to classify patients with malnutrition, reducing the bias of weight overestimation caused by tumor mass in this population.

Even nutrition risk classification using BMI‐for‐age (*z* score <−1) showed a lower prevalence than nutrition depletion as indicated by MUAC and CC. The fifth to 15th percentile range of MUAC, which is used to define nutrition risk, was not applied in this study because the focus was on the established malnutrition cutoffs used at the IOP/GRAACC. However, this approach could be used in future research to more sensitively assess nutrition risk. In this study, low food intake was independently associated with malnutrition when measured by more sensitive parameters, such as MUAC and CC. The persistence of this association may have been influenced by sample size when other factors were included in the statistical model.

The nutritionist's subjective perception of nutrition status was the most sensitive predictor of malnutrition. Experienced nutritionists can detect subtle nutrition changes that quantitative methods often miss, especially in children, in whom anthropometry may fail to detect the early stages of malnutrition. Research also suggests that subjective assessment tools are useful for populations with conditions such as renal failure and cancer because conventional measures, such as weight and height, often underestimate malnutrition in these populations.^33^, ^34^

The nutrition risk stratification may vary depending on the tool used. For example, De Oliveira Canedo et al found that STRONGkids identified a 20% prevalence of high nutrition risk, whereas the SCAN tool identified a 45% prevalence of nutrition risk in a similar sample.^35^ NUTRICCAN identified a higher percentage of patients at moderate or high risk (80.2%) compared with SCAN's identification of nutrition risk (51.3%). Stratifying patients into multiple risk levels allows for better allocation of resources and prioritization of nutrition interventions, especially in settings with limited resources.^36^

De Oliveira Canedo et al reported a low sensitivity (37.5%) of SCAN in predicting malnutrition at diagnosis and throughout treatment as measured by anthropometric parameters. However, the sensitivity increased to 87.5% when risk was considered throughout treatment.^35^ In our study, the sensitivity of SCAN in predicting malnutrition at any time during treatment was lower (65.2%) than in their paper. However, the cross‐sectional design of the study limited the predictive analysis of the tool because the observed outcome was malnutrition detected at admission rather than malnutrition assessed over time.

Ideally, a nutrition screening tool would predict the risk of adverse nutrition outcomes before malnutrition is diagnosed. Thus, it is necessary to examine the risk measured by NUTRICCAN in a cohort that evaluates clinical and anthropometric outcomes from diagnosis to treatment. The findings should be interpreted with caution because of the limited sample size, which may have affected the strength of the associations identified by statistical testing. Additionally, the results may not be generalizable to the entire pediatric oncology population because the sample was collected from a single treatment center. Because this tool incorporates some socioeconomic factors, its applicability may be country specific or region specific, necessitating validation in different settings before broader use. Future validation in larger cohorts with longitudinal follow‐up is essential to better evaluate its predictive ability regarding clinical and nutrition outcomes throughout treatment.

Nevertheless, this study highlights the importance of a tailored nutrition screening tool for the pediatric oncology population that takes into account its clinical characteristics and the Brazilian socioeconomic context. NUTRICCAN outperformed SCAN and STRONGkids in categorizing nutrition risk into more specific levels in this sample. This could lead to more targeted and effective management, particularly in settings with limited resources.

## AUTHOR CONTRIBUTIONS

Cristiane Ferreira Marçon and Fernanda Luisa Ceragioli Oliveira contributed equally to the conception and design of the study. Fernanda Luisa Ceragioli Oliveira, Cristiane Ferreira Marçon, and Carolina Araújo dos Santos contributed to the acquisition and analysis of the data. Cristiane Ferreira Marçon and Fernanda Luisa Ceragioli Oliveira contributed to the interpretation of the data. Cristiane Ferreira Marçon, Carolina Araújo dos Santos, and Fernanda Luisa Ceragioli Oliveira drafted the manuscript. All authors critically revised the manuscript, agree to take full responsibility for the integrity and accuracy of the work, and read and approved the final manuscript.

## CONFLICT OF INTEREST STATEMENT

None declared.

## Supporting information

NUTRICCAN ‐ English.

NUTRICCAN ‐ Portuguese.

Supplementary Tables 1 to 4 ‐ Odd Ratio.
