# Supplementary material for: Development and validation of a nutrition risk screening for patients with childhood cancer in Brazil (NUTRICCAN)
Source: Nutr Clin Pract. 2025 Dec 4;41(3):768–78. doi: 10.1002/ncp.70076 (PMC13193373; doi:10.1002/ncp.70076)
Supplement: Supplementary file 1 — NUTRICCAN ‐ English. [file NCP-41-768-s002.pdf]

NUTRICCAN – Nutritional Risk Screening for Childhood Cancer

Name: ____

Date of Birth: ___/___/___ Hospital ID: ___________________________

Admission Date: ___/___/___ Screening Date: ___/___/___ Rescreening Date: ___/___/___

Assessed by: ____________________________________ Department: ____________________________________

1) Which group best describes the patient's oncological diagnosis?

Tumors with extensive abdominal masses<br>*e.g., Wilms tumor stage III/IV, neuroblastoma stage III/IV, abdominal/pelvic rhabdomyosarcoma, Burkitt-type non-Hodgkin lymphoma (NHL) in the abdominal region, among others*<br>Tumors in the head and neck region (at any stage)<br>*e.g., Head/neck carcinomas (oral cavity, larynx, pharynx, esophagus), rhabdomyosarcomas (oral cavity, larynx, pharynx, esophagus), non-Hodgkin lymphoma (NHL) in the head and neck, among others.*<br>Medulloblastoma<br>Diencephalic tumors<br>Ewing's sarcoma<br>Osteosarcoma<br>Relapse of leukemia/lymphoma or with comorbidities (e.g., pancreatitis)	Other solid tumors<br>*e.g., Wilms tumor stage I/II, neuroblastoma stage I/II, etc.*<br>Acute leukemia or lymphoma without complications	Disease in remission during maintenance therapy<br><br>Craniopharyngioma
3 points	2 points	1 point

2) Is the current treatment intensive and may lead to nutritional impairment? If any of the items are true, score:

• First cycle of chemotherapy<br>• Recent use (within past 15 days) or planned use for the coming days, of chemotherapeutic agents with emetic potential or intestinal toxicity:<br>*e.g., cisplatin, cyclophosphamide, methotrexate, cytarabine, fluorouracil, irinotecan*<br>• Treatments involving (in the last 3 months or planned for the next 7 days):<br>*Head and neck surgeries (except neurosurgery) or abdominal surgeries*<br>*Pelvic, abdominal, head and neck, or cranial radiotherapy (with or without neuroaxis)*<br>• Postoperative complications (consider up to 15 days after surgery)<br>• Post-hematopoietic stem cell transplant complications (e.g., graft-versus-host disease [GVHD])	3 points

3) Does the patient present any of the risk factors below? Score each true item:

Age group: Infants under 1 year old	3 points
Age group: Early childhood (1 to 3 years) or adolescence (rapid growth phase)	2 points
Low socioeconomic status (<1 minimum wage) or low caregiver education (<8 years of schooling)	1 point
Pain (regardless of location) that interferes with food intake	1 point
Respiratory discomfort that interferes with food intake	1 point
Hospitalization duration longer than 15 days	1 point
Readmission within 7 days	1 point
SCORE	

4) Does the patient have gastrointestinal alterations in the last 24 hours? Score each true item:

Diarrhea: 5 or more liquid stools in the last 24 hours OR any frequency with clinical repercussions (e.g., dehydration)	2 points
Mucositis: Presence of oral mucositis that, regardless of grade, hinders food intake	2 points

Vomiting: 3 or more episodes of vomiting in the last 24 hours OR any frequency with clinical repercussions (e.g., dehydration)	2 points
SCORE	

5) Has the patient experienced changes in their food intake in the last few days? Score each true item:

Oral or enteral fasting ≥ 72 hours without nutritional support (enteral or parenteral)	5 points
Anorexia: Complete lack of appetite in the last 48 hours	3 points
Less than 2 complete meals (about 50% of daily needs) in the last 3 days **OR**<br>Regular food intake (about 70% of daily needs) for one week or more **OR**<br>Use of enteral feeding tube with intake below the prescribed volume	2 points
Not applicable (fasting for examination/surgery OR full enteral diet administration)	0 points

6) Do the parents/caregivers (or the patient themselves) notice that the patient has lost weight in the last month?

Yes, there was significant and very noticeable weight loss	5 points
Yes, there was slight or mild weight loss (e.g., clothes became looser)	3 points
Not sure if weight was lost	1 point
Did not lose weight	0 points

7) Does the patient show signs of nutritional compromise based on the overall patient assessment?

The patient shows clear signs of muscle and/or fat depletion, cachexia, sarcopenia, or a record of weight loss >2% documented in the medical record, leaving no doubt about the presence of malnutrition	10 points
I have doubts whether the patient shows nutritional compromise (due to hyperhydration, for example)	5 points

**FINAL SCORE**	

CLASSIFICATION AND FOLLOW-UP:

1–4 points: Low risk	5–9 points: Intermediate risk	≥10 points: High risk
Repeat screening in 7 days. Full nutritional assessment every 30 days.	Repeat screening in 3 days. Full nutritional assessment every 15 days.	Full nutritional assessment every 7 days. Refer to Nutritional Team. Daily food intake monitoring for 7 days. Rescreen in 7 days.
