# Supplementary material for: Development and validation of a nutrition risk screening for patients with childhood cancer in Brazil (NUTRICCAN)
Source: Nutr Clin Pract. 2025 Dec 4;41(3):768–78. doi: 10.1002/ncp.70076 (PMC13193373; doi:10.1002/ncp.70076)
Supplement: Supplementary file 2 — NUTRICCAN ‐ Portuguese. [file NCP-41-768-s003.pdf]

NUTRICCAN - TRIAGEM DE RISCO NUTRICIONAL PARA CRIANÇAS E ADOLESCENTES COM CÂNCER

Nome: ____

Data de Nascimento: ___/___/___ Registro hospitalar: _________________________

Data da internação: ___/___/___ Data da triagem: ___/___/___ Data da retriagem: ___/___/___

Aplicado por: __________________________________ Setor: __________________________________

1) Qual grupo melhor descreve o diagnóstico oncológico do paciente?

Tumores com massas abdominais extensas<br>*Exemplos: Tumor Wilms - estadio III e IV, neuroblastoma - estadio III e IV, rabdomiossarcomas abdominais ou pélvicos, linfoma não Hodgkin (LNH) do tipo burkitt de região abdominal, entre outros.*<br>Tumores em região de cabeça e pescoço (em qualquer estágio)<br>*Exemplos: Carcinomas de cabeça/pescoço (cavidade oral, laringe, faringe, esôfago), rabdomiossarcomas (cavidade oral, laringe, faringe, esôfago), linfoma não Hodgkin (LNH) de cabeça e pescoço, entre outros.*<br>Meduloblastoma<br>Tumores diencefálicos<br>Sarcoma de Ewing<br>Osteossarcoma<br>Recaídas de leucemias e linfomas ou com comorbidades (ex. pancreatite)	Outros tumores sólidos<br>*Exemplos: Tumor Wilms - estadio I e II, neuroblastoma – estádio I e II, entre outros*<br>Leucemias agudas ou linfomas sem complicações	Doenças em remissão, durante o tratamento de manutenção<br>Craniofaringioma
3 pontos	2 pontos	1 ponto

2) O tratamento atual é intensivo e pode levar a comprometimento nutricional? Se alguns dos itens forem verdadeiros, pontuar:

1º ciclo de quimioterapia;<br>Uso recente (nos últimos 15 dias) ou com programação para os próximos dias, de quimioterápicos com potencial emético ou com toxicidade intestinal:<br>• Ex. Cisplatina, Ciclofosfamida, Metotrexato, Citarabina, Fluorouracila, Irinotecano<br>Tratamentos que envolvam (nos últimos 3 meses ou com programação para os próximos 7 dias):<br>• Cirurgias de cabeça e pescoço (exceto neurocirurgia) ou cirurgias abdominais<br>• Radioterapia pélvica, abdominal, cabeça e pescoço ou crânio (com ou sem neuroeixo)<br>Complicações pós-operatórias (considerar até 15 dias após a cirurgia);<br>Complicações pós-transplante de células hematopoiéticas (ex. DECH)	3 pontos

3) O paciente apresenta algum dos fatores de risco abaixo? Pontuar cada um dos itens verdadeiros:

Faixa etária: Lactentes com menos de 1 ano completo	3 pontos
Faixa etária: Primeira infância (1 a 3 anos) ou adolescência (crescimento acelerado)	2 pontos
Baixas condições socioeconômicas (<1 salário-mínimo) **OU** baixa escolaridade dos cuidadores (<8 anos de estudo)	1 ponto
Dor (independentemente do local) que interfere na aceitação alimentar	1 ponto
Desconforto respiratório, que interfere na aceitação alimentar	1 ponto
Tempo de internação maior de 15 dias	1 ponto
Reinternação em menos de 7 dias	1 ponto
SOMATÓRIA	

4) O paciente apresenta alterações gastrointestinais nas últimas 24 horas? Pontuar cada um dos itens verdadeiros:

Diarreia: 05 ou mais evacuações líquidas nas últimas 24 horas **OU** qualquer frequência com repercussão clínica (por exemplo: desidratação)	2 pontos
Mucosite: Presença de mucosite oral que, independentemente do grau, dificulte a alimentação	2 pontos
Vômitos: 03 ou mais episódios de vômito nas últimas 24 horas **OU** qualquer frequência com repercussão clínica (por exemplo: desidratação)	2 pontos
SOMATÓRIA	

5) O paciente apresenta alterações da sua alimentação nos últimos dias? Se alguns dos itens forem verdadeiros, pontuar:

Jejum via oral ou enteral ≥ 72 horas, sem suporte nutricional (enteral ou parenteral)	5 pontos
Anorexia: Inapetência completa nas últimas 48 horas	3 pontos
Menos de 2 refeições completas (cerca de 50% das suas necessidades diárias) nos últimos 3 dias **OU** Aceitação alimentar regular (cerca de 70% das suas necessidades diárias) por uma semana ou mais **OU** Uso de sonda enteral com oferta abaixo do volume prescrito	2 pontos
Não se aplica (jejum para exame/cirurgia OU oferta plena de dieta via enteral)	0 pontos

6) Os pais/cuidadores (ou o próprio paciente) percebem que o paciente emagreceu no último mês?

Sim, teve emagrecimento grave e muito perceptível	5 pontos
Sim, teve pouco ou leve emagrecimento (por exemplo, roupas ficaram mais largas)	3 pontos
Não tem certeza se emagreceu	1 ponto
Não emagreceu	0 pontos

7) O paciente apresenta comprometimento do estado nutricional considerando a avaliação global do paciente?

O paciente apresenta sinais claros de depleção muscular e/ou adiposa, caquexia, sarcopenia ou registro de perda de peso >2% registrado em prontuário, que não deixam dúvidas quanto à presença de desnutrição	10 pontos
Tenho dúvidas se o paciente apresenta comprometimento do estado nutricional (devido hiperhidratação, por exemplo)	5 pontos

**PONTUAÇÃO FINAL**	

CLASSIFICAÇÃO E SEGMENTO:

Risco baixo - 1 a 4 pontos	Risco intermediário - 5 a 9 pontos	Risco alto - ≥10 pontos
Reaplicar nova triagem em 7 dias. Realizar avaliação nutricional completa a cada 30 dias.	Reaplicar nova triagem em 3 dias. Realizar avaliação nutricional completa a cada 15 dias.	Realizar avaliação nutricional completa a cada 7 dias e encaminhar para seguimento com EMTN. Acompanhar diariamente o paciente nos próximos 7 dias com avaliação de aceitação alimentar diária. Reaplicar nova triagem em 7 dias.
