# Supplementary material for: Development and validation of a nutrition risk screening for patients with childhood cancer in Brazil (NUTRICCAN)
Source: Nutr Clin Pract. 2025 Dec 4;41(3):768–78. doi: 10.1002/ncp.70076 (PMC13193373; doi:10.1002/ncp.70076)
Supplement: Supplementary file 3 — Supplementary Tables 1 to 4 ‐ Odd Ratio. [file NCP-41-768-s001.docx]

Supplementary Table **1. Multivariate Logistic Regression Analysis of NUTRICCAN Variables Associated with Malnutrition Defined by BMI-for-Age < -2 SD**

|  | **BMI/Age <-2 z-score** | | | |
| --- | --- | --- | --- | --- |
|  | **Univariate Analysis** | | **Multivariate Analysis** | |
|  | **OR (95% CI)** | **p** | **OR (95% CI)** | **p** |
| **Item 1** |  |  |  |  |
| **1.1–1.2** | 1,50 (0,4-5,2) | 0,52 | 1,78 (0,4-8,6) | 0,47 |
| **1.1–1.3** | 4,3e+6 (0,0-inf) | 0,99 | 4,3e+7 (0,0-inf) | 0,99 |
| **1.2–1.3** | 2,9e+6 (0,0-inf) | 0,99 | 2,4e+7 (0,0-inf) | 0,99 |
| **Item 2** | 0,53 (0,1-1,9) | 0,33 | 0,87 (0,9-4,1) | 0,86 |
| **Item 3** | 2,9e-7 (0,0-inf) | 0,99 | 1,2e-8 (0,0-inf) | 0,99 |
| **Item 4** | 1,09 (0,3-3,7) | 0,88 | 0,84 (0,9-3,8) | 0,82 |
| **Item 5** | 1,87 (0,5-6,8) | 0,34 | 3,63 (0,7-19,7) | 0,13 |
| **Item 6** | 1,47 (0,2-12,7) | 0,73 | 3,98 (0,2-94,4) | 0,39 |
| **Item 7** | 2,9e-7 (0,0-inf) | 0,99 | 5,3e-8 (0,0-inf) | 0,99 |
| **Item 8** | 1,91 (0,2-17,1) | 0,56 | 6,00 (0,3-122,2) | 0,24 |
| **Item 9** | 2,8e-7 (0,0-inf) | 0,99 | 9,2e-9 (0,0-inf) | 0,99 |
| **Item 10** | 8,1e-7 (0,0-inf) | 0,99 | 4,9e-8 (0,0-inf) | 0,99 |
| **Item 11** | 6,95 (0,6-83,4) | **0,13** | 8,20 (0,2-346,6) | 0,27 |
| **Item 12** | 2,9e-7 (0,0-inf) | 0,99 | 4,4e-7 (0,0-inf) | 0,99 |
| **Item 13** |  |  |  |  |
| **13.3–13.4** | 0,57 (0,1-2,2) | 0,42 | 0,17 (0,0-1,0) | **0,04** |
| **Item 14** |  |  |  |  |
| **14.1–14.2** | 1,5e-7 (0,0-inf) | 0,99 | 4,3e-9 (0,0-inf) | 0,99 |
| **14.1–14.3** | 3,1e-7 (0,0-inf) | 0,99 | 8,5e-9 (0,0-inf) | 0,99 |
| **14.2–14.3** | 2,03 (0,2-19,1) | 0,54 | 2,00 (0,1-28,1) | 0,60 |
| **14.1–14.4** | 4,2e-7 (0,0-inf) | 0,99 | 7,1e-9 (0,0-inf) | 0,99 |
| **14.2–14.4** | 2,81 (0,8-10,4) | **0,12** | 1,70 (0,3 – 9,4) | 0,56 |
| **14.3–14.4** | 1,38 (0,1-12,8) | 0,77 | 0,83 (0,1-11,8) | 0,89 |
| **Item 15** |  |  |  |  |
| **15.1-NA** | 15,60 (1,9-127,4) | **0,01** | 41,00 (3,1-535,2) | **<0,01** |
| **15.2-NA** | 2,45 (0,1-40,5) | 0,53 | 3,60 (0,1-128,5) | 0,48 |
| **15.1–15.2** | 6,36 (0,8-53,0) | **0,09** | 11,30 (0,6-204,1) | 0,10 |

NA – Not applicable
e – Exponential
inf – Infinite

**Supplementary Table 2. Multivariate Logistic Regression Analysis of NUTRICCAN Variables Associated with Malnutrition Defined by MUAC Below the 5th Percentile**

|  | **MUAC < 5th percentile** | | | |
| --- | --- | --- | --- | --- |
|  | **Univariate Analysis** | | **Multivariate Analysis** | |
|  | **OR (95% CI)** | **p** | **OR (95% CI)** | **p** |
| **Item 1** |  |  |  |  |
| **1.1–1.2** | 1,14 (0,5-2,4) | 0,34 | 1,29 (0,4-3,8) | 0,65 |
| **1.1–1.3** | 1,06 (0,2-5,8) | 0,94 | 1,48 (0,1-16,6) | 0,75 |
| **1.2–1.3** | 0,93 (0,2-5,0) | 0,93 | 1,15 (0,1-12,3) | 0,90 |
| **Item 2** | 0,35 (0,2-0,8) | **<0,01** | 0,22 (0,1-0,70) | **0,01** |
| **Item 3** | 5,2e-7 (0,0-inf) | 0,99 | 5,3e-7 (0,0-inf) | 0,99 |
| **Item 4** | 0,60 (0,3-1,3) | **0,20** | 0,54 (0,2-1,5) | 0,26 |
| **Item 5** | 1,98 (0,9-4,5) | **0,10** | 2,02 (0,6-6,4) | 0,23 |
| **Item 6** | 3,34 (0,9-12,3) | **0,07** | 15,70 (2,2-112,9) | **<0,01** |
| **Item 7** | 1,01 (0,1-10,0) | 0,99 | 0,52 (0,0-11,7) | 0,68 |
| **Item 8** | 0,49 (0,0-4,2) | 0,52 | 0,50 (0,0-8,7) | 0,63 |
| **Item 9** | 1,56 (0,4-6,6) | 0,54 | 1,48 (0,1-14,9) | 0,74 |
| **Item 10** | 3,08 (0,2-50,6) | 0,43 | 0,83 (0,0-61,0) | 0,93 |
| **Item 11** | 1,53 (0,1-17,3) | 0,73 | 3,70 (0,1-159,0) | 0,50 |
| **Item 12** | 0,75 (0,1-6,9) | 0,80 | 1,34 (0,0-82,5) | 0,89 |
| **Item 13** |  |  |  |  |
| **13.3–13.4** | 1,64 (0,8-3,5) | **0,20** | 0,73 (0,2-2,2) | 0,58 |
| **Item 14** |  |  |  |  |
| **14.1–14.2** | 0,62 (0,1-6,0) | 0,68 | 0,22 (0,0-3,6) | 0,29 |
| **14.1–14.3** | 1,10 (0,1-15,1) | 0,94 | 0,37 (0,0-11,1) | 0,57 |
| **14.2–14.3** | 1,77 (0,3-9,5) | 0,51 | 1,65 (0,2-15,9) | 0,66 |
| **14.1–14.4** | 0,54 (0,1-4,9) | 0,59 | 0,04 (0,0-0,7) | **0,03** |
| **14.2–14.4** | 0,87 (0,4-2,1) | 0,76 | 0,19 (0,1-0,7) | **0,02** |
| **14.3–14.4** | 0,49 (0,1-2,4) | 0,38 | 0,11 (0,0-1,1) | 0,06 |
| **Item 15** |  |  |  |  |
| **15.1-NA** | 12,40 (4,5-34,0) | **<0,01** | 29,20 (7,8-110,1) | **<0,01** |
| **15.2-NA** | 2,39 (0,7-8,6) | **0,18** | 3,71 (0,7-18,7) | 0,11 |
| **15.1–15.2** | 5,20 (1,7-15,9) | **<0,01** | 7,88 (1,7-35,5) | **<0,01** |

NA – Not applicable
e – Exponential
inf – Infinite

**Supplementary Table 3. Multivariate Logistic Regression Analysis of NUTRICCAN Variables Associated with Malnutrition Defined by Calf Circumference Below Age- and Sex-Specific Cutoff**

|  | **CC < cutoff point** | | | |
| --- | --- | --- | --- | --- |
|  | **Univariate Analysis** | | **Multivariate Analysis** | |
|  | **OR (95% CI)** | **p** | **OR (95% CI)** | **p** |
| **Item 1** |  |  |  |  |
| **1.1–1.2** | 0,88 (0,4-1,6) | 0,58 | 0,87 (0,4-1,9) | 0,74 |
| **1.1–1.3** | 2,27 (0,4-11,9) | 0,33 | 1,59 (0,2-10,7) | 0,63 |
| **1.2–1.3** | 1,87 (0,3-10,0) | 0,46 | 1,82 (0,3-11,7) | 0,53 |
| **Item 2** | 0,73 (0,4-1,4) | 0,34 | 0,70 (0,3-1,5) | 0,37 |
| **Item 3** | 6,32 (0,7-58,0) | **0,10** | 8,70 (0,7-113,4) | 0,10 |
| **Item 4** | 0,95 (0,5-1,8) | 0,87 | 1,67 (0,7-3,8) | 0,22 |
| **Item 5** | 2,46 (1,2-5,2) | **0,02** | 2,45 (0,9-6,3) | 0,06 |
| **Item 6** | 0,99 (0,3-3,7) | 0,99 | 1,03 (0,2-5,2) | 0,97 |
| **Item 7** | 4,65 (0,5-45,8) | **0,19** | 1,03 (0,1-13,4) | 0,98 |
| **Item 8** | 1,53 (0,4-6,3) | 0,56 | 1,93 (0,3-10,5) | 0,48 |
| **Item 9** | 0,73 (0,2-3,0) | 0,67 | 0,63 (0,1-3,6) | 0,60 |
| **Item 10** | 2,5e-7 (0,0-inf) | 0,99 | 6,0e-8 (0,0-inf) | 0,99 |
| **Item 11** | 0,74 (0,1-8,4) | 0,81 | 0,94 (0,1-14,7) | 0,97 |
| **Item 12** | 0,36 (0,0-3,3) | 0,37 | 1,48 (0,1-19,5) | 0,77 |
| **Item 13** |  |  |  |  |
| **13.3–13.4** | 2,07 (1,1-4,0) | **0,03** | 1,94 (0,8-4,6) | 0,13 |
| **Item 14** |  |  |  |  |
| **14.1–14.2** | 2,35 (0,4-14,5) | 0,36 | 1,94 (0,3-14,1) | 0,51 |
| **14.1–14.3** | 3,60 (0,5-27,1) | 0,21 | 1,94 (0,2-18,7) | 0,57 |
| **14.2–14.3** | 1,53 (0,4-5,4) | 0,51 | 1,00 (0,2-4,5) | 0,99 |
| **14.1–14.4** | 3,43 (0,6-19,7) | **0,17** | 1,53 (0,2-10,7) | 0,67 |
| **14.2–14.4** | 1,46 (0,7-3,1) | 0,33 | 0,79 (0,3-2,0) | 0,63 |
| **14.3–14.4** | 0,95 (0,3-3,1) | 0,93 | 0,79 (0,2-3,4) | 0,75 |
| **Item 15** |  |  |  |  |
| **15.1-NA** | 4,80 (2,2-10,6) | **<0,01** | 4,52 (1,8-11,4) | **<0,01** |
| **15.2-NA** | 4,00 (1,6-9,9) | **<0,01** | 3,43 (1,1-10,3) | **0,03** |
| **15.1–15.2** | 1,21 (0,5-3,0) | 0,68 | 1,32 (0,4-3,8) | 0,61 |

NA – Not applicable
e – Exponential
inf – Infinite

**Supplementary Table 4. Multivariate Logistic Regression Analysis of NUTRICCAN Variables Associated with the Presence of at Least One Altered Anthropometric Parameter**

|  | **Malnutrition (any criterion)** | | | |
| --- | --- | --- | --- | --- |
|  | **Univariate Analysis** | | **Multivariate Analysis** | |
|  | **OR (95% CI)** | **p** | **OR (95% CI)** | **p** |
| **Item 1** |  |  |  |  |
| **1.1–1.2** | 0,73 (0,4-1,4) | 0,35 | 0,72 (0,3-1,6) | 0,44 |
| **1.1–1.3** | 1,18 (0,3-5,4) | 0,83 | 1,03 (0,2-6,5) | 0,97 |
| **1.2–1.3** | 1,62 (0,4-7,3) | 0,52 | 1,43 (0,2-8,5) | 0,69 |
| **Item 2** | 0,59 (0,3-1,1) | **0,11** | 0,60 (0,3-1,3) | 0,21 |
| **Item 3** | 5,05 (0,5-46,2) | **0,15** | 6,50 (0,4-104,8) | 0,19 |
| **Item 4** | 0,72 (0,4-1,4) | 0,33 | 1,15 (0,5-2,6) | 0,74 |
| **Item 5** | 2,87 (1,3-6,2) | **<0,01** | 2,84 (1,1-7,6) | **0,04** |
| **Item 6** | 1,88 (0,5-6,9) | 0,34 | 2,15 (0,4-11,0) | 0,35 |
| **Item 7** | 3,73 (0,4-36,7) | 0,26 | 0,98 (0,1-13,5) | 0,99 |
| **Item 8** | 1,21 (0,3-5,0) | 0,79 | 1,70 (0,3-10,2) | 0,56 |
| **Item 9** | 0,58 (0,1-2,4) | 0,46 | 0,36 (0,1-2,4) | 0,29 |
| **Item 10** | 1,21 (0,1-19,6) | 0,89 | 0,18 (0,0-8,3) | 0,38 |
| **Item 11** | 0,60 (0,1-6,7) | 0,67 | 0,97 (0,1-16,2) | 0,98 |
| **Item 12** | 0,80 (0,1-4,9) | 0,80 | 3,19 (0,3-34,1) | 0,34 |
| **Item 13** |  |  |  |  |
| **13.3–13.4** | 2,01 (1,0-3,9) | **0,04** | 1,39 (0,6-3,4) | 0,46 |
| **Item 14** |  |  |  |  |
| **14.1–14.2** | 1,89 (0,3-11,6) | 0,49 | 1,15 (0,1-8,6) | 0,89 |
| **14.1–14.3** | 3,60 (0,5-27,1) | 0,21 | 1,64 (0,2-16,2) | 0,67 |
| **14.2–14.3** | 1,90 (0,5-6,7) | 0,32 | 1,42 (0,3-6,7) | 0,66 |
| **14.1–14.4** | 2,63 (0,5-15,1) | 0,28 | 0,67 (0,1-4,9) | 0,70 |
| **14.2–14.4** | 1,39 (0,6-3,0) | 0,40 | 0,59 (0,2-1,6) | 0,29 |
| **14.3–14.4** | 0,73 (0,2-2,3) | 0,60 | 0,41 (0,1-1,8) | 0,25 |
| **Item 15** |  |  |  |  |
| **15.1-NA** | 7,55 (3,3-17,0) | **<0,01** | 9,93 (3,6-26,9) | **<0,01** |
| **15.2-NA** | 4,23 (1,7-10,4) | **<0,01** | 3,83 (1,2-11,7) | **0,02** |
| **15.1–15.2** | 1,80 (0,7 – 4,6) | 0,23 | 2,59 (0,8-8,0) | 0,10 |

NA – Not applicable
e – Exponential
inf – Infinite
